# Compliance Checks Decrease Cigarette Sales Rates to Pseudo-Underaged Mystery Shoppers: A Quasi-Experimental Control Group Study

**DOI:** 10.3390/ijerph192013161

**Published:** 2022-10-13

**Authors:** Kristin Feltmann, Johanna Gripenberg, Tobias H. Elgán

**Affiliations:** STAD (Stockholm Prevents Alcohol and Drug Problems), Centre for Psychiatry Research, Department of Clinical Neuroscience, Karolinska Institute and Stockholm Health Care Services, Region Stockholm, 11364 Stockholm, Sweden

**Keywords:** compliance checks, mystery shopping, cigarettes, age limits, tobacco law, youth, ID control

## Abstract

To control adherence to age limits regarding sales of tobacco products, Swedish authorities can conduct compliance checks. Compliance checks involve prior information to all retailers, mystery shopping, and subsequent feedback to the retailers. This study investigated whether compliance checks can decrease the rates of cigarette sales to underaged adolescents. Test purchases of cigarettes were conducted using pseudo-underaged mystery shoppers, i.e., 18-year-old adolescents with a younger appearance not carrying ID, to measure the refusal rate and rate of ID checks. Test purchases were conducted at 257 retail outlets in 13 municipalities in Stockholm County at baseline 2017 and follow-up 2019, respectively. In between the measurements, six municipalities (intervention area) conducted compliance checks, and seven municipalities were used as a comparison. Comparing baseline and follow-up, rates of refusal (70.4 to 95.8%) and ID checks (80.3 to 95.8%) improved in the intervention area. In the comparison area, refusal rates increased (80.9 to 85.2%), and ID check rates remained stable (at 86.1%). Significant group × time interaction effects reveal that the rates of refusal and ID checks differently changed in the study areas over time. These results indicate that compliance checks are an effective method to decrease cigarette sales to underaged adolescents.

## 1. Introduction

Tobacco smoking contributes to a number of diseases, including cardiovascular diseases and various forms of cancer, thereby causing a serious public health problem [1,2]. Although smoking has been declining globally over time, as of 2019 it was still the second leading risk factor for disability adjusted life-years (DALYs) [3]. Adolescents are a particularly vulnerable group as smoking during adolescence increases the risk of remaining a smoker as an adult [4,5]. To reduce the prevalence of smoking among adolescents, a variety of interventions have been investigated, including raising taxes on tobacco products, plain packaging, health warnings, smoking cessation support using mass media campaigns and technology, as well as preventive efforts in the school setting [6,7,8]. Studies have also shown that prevention efforts and policy changes that reduce the availability and sales rates of cigarettes to adolescents were effective strategies to reduce smoking prevalence among adolescents [9,10,11,12].

The majority of studies investigating strategies and interventions to reduce sales of tobacco products to adolescents have been conducted in the U.S. and Australia (for a literature review, see Cochrane review) [13]. For example, education of sales clerks, information campaigns targeting society, and warnings or sanctions (such as fines or sentences) to merchants selling tobacco products to underage customers were evaluated. Many of the studies demonstrated a decrease in sales rates to adolescents after implementing various intervention strategies; however, several studies could not draw conclusions about causality because they did not include a comparison group (for a literature review, see Cochrane review) [13]. Some of the studies that included a comparison group could show a faster or larger decrease in sales rates in the intervention compared to the comparison group [14,15,16,17]. However, other studies showed a similar decrease in sales rates in both groups [11,18,19]. In general, interventions that included enforcement, such as fines or sales bans, were effective in decreasing sales of tobacco to adolescents [9,12,13,20,21,22]. Furthermore, interventions that did not consist of enforcement, but of information to local society and retailers through the media, letters, or dialogues, have also proven effective [14,16,23,24,25,26,27]. Overall, interventions using enforcement and multicomponent educational strategies seem to be more effective than interventions using information to retailers alone [13,28]. Today, performing compliance checks is an established method of control, and is conducted routinely by the FDA in the U.S. These inspections include mystery shopping and enforcement upon violations, such as warnings, fines, or no-tobacco-sales orders. An analysis of over 136,000 FDA-compliance checks in 2015 showed that a higher retailer violation rate was associated with a higher youth smoking prevalence, and that higher taxes and more prevention efforts reduced the likelihood of retailer violations [29]. 

In 2019, the age limit for the sale of tobacco products was raised to 21 years in all states in the U.S. due to a “Tobacco 21” law. However, several states and communities had already raised the age limit to 21 before 2019. A survey among 16,000 students in Massachusetts demonstrated that both retail purchases of cigarettes and smoking prevalence reduced among adolescents in a local community, where the age limit had been raised in 2005, but not in 16 control communities that had not raised the age limit [30]. In New York City, using female mystery shoppers, the rate of ID checks declined from 71% before to 62% after the age limit had been raised in 2014. In the study, compliance rates were lower in independent stores, compared to chain-stores [31]. Another study in California, using 15 to 16-year-old mystery shoppers, showed that the retailer violation rate had decreased from 10% to 6% following the implementation of Tobacco 21 in 2016 [32]. Moreover, when mystery shoppers conducted test purchases in Columbus, Ohio, there was an increase in ID checks from 39% in 2017, to 78% in 2018 when the law had been enacted. An analysis of records of municipality-conducted compliance checks revealed that ID checks had been 74% immediately after the Tobacco 21 enforcement, but declined to 62% during the subsequent year [33]. 

According to the global tobacco survey, in many European countries a large proportion of 13 to 15-year-old smokers reported to have purchased cigarettes from commercial sources and had never been refused when attempting to buy during the previous 30 days [34]. Despite this need to reduce underaged purchases, there are few studies in Europe in recent years regarding age controls and related interventions. For example, a recent study conducted in Portugal revealed that 95% of adolescents reported to be able to buy cigarettes and argued that age controls are generally weakly enforced [35]. Another study in France showed that in 65% of purchase attempts 17-year-olds were sold cigarettes [36]. A study conducted in the Netherlands using 15-year-old mystery shoppers showed that using face scanners at vending machines led to an almost complete compliance to ID checks, in contrast to regular in store purchase attempts where ID was only requested in 12% of the cases [37]. These studies indicate that compliance with age laws is relatively low in Europe, and is not widely enforced. In addition, there are European studies arguing that stricter enforcement of age limits might not necessarily reduce smoking rates. For example, a study analyzing bans from 1990 to 2016 in Switzerland stated that effects on smoking prevalence was marginal and that adolescents circumvented bans by buying through peers [38]. Similarly, a survey and focus-group study conducted in seven European countries revealed that adolescents buy cigarettes from non-compliant retailers (smaller shops and cafés), or through other people in countries where enforcement was high [39]. 

In Sweden, the age limit of 18-years old was implemented in 1997. According to the Swedish Tobacco Act (1993:581; 2018:2088) anyone selling tobacco products must ensure that the customer is 18 years or older, usually by checking ID. Retailers must also display a clearly visible 18-year age-limit sign. Retailer compliance with the legislation on the age limit is necessary to achieve the preventive effect on smoking. The tobacco control authority within each municipality, together with the police, are responsible for monitoring this compliance. Municipalities should regularly visit the outlets to monitor the display of age-limit signs, retailer promotion, and the placement of tobacco products and, ideally, also conduct compliance checks. A guide with a compliance check protocol is provided by the Public Health Agency of Sweden, which includes: (1) informing all retailers that compliance checks will be conducted and reminding them of the current legislation, (2) performing mystery shopping with pseudo-underaged shoppers (18-years old with a younger appearance), (3) having a dialogue with the sales clerk and store manager regarding the outcome of the purchase attempt, as well as the legislation and routines for age controls, and (4) informing the store owner about the purchase attempt. However, the results of compliance checks cannot be used by the municipalities as a basis for sanctions. In Sweden, compliance checks are used on a more or less regular basis in several, but not all, municipalities; however, they have not been thoroughly evaluated. A Swedish study used tobacco test purchases, which are performed secretively and involve no feedback, to assess the sales rates to underage adolescents before and up to eight years after the age limit was implemented in 1997 [40]. Between 1996 and 2005, the refusal rates of cigarette sales to pseudo-underaged mystery shoppers increased in all three areas tested in Sweden from 16% to 52%, which the authors suggested were due to activities (e.g., monitoring visits) conducted by the authorities [40]. The aim of the current study is to evaluate the outcomes of compliance checks, involving only feedback and no sanctions, in terms of increasing ID checks and reducing cigarette sales rates to adolescents.

## 2. Materials and Methods

### 2.1. Design

This is a quasi-experimental control group study using a repeated cross-sectional design. Covert test purchases at outlets selling cigarettes were conducted to estimate the rates of ID checks and sales of cigarettes to pseudo-underaged adolescents at baseline (June 2017) and follow-up (June 2019). 

### 2.2. Setting

Municipalities in Stockholm County where compliance checks had not been performed within the two years preceding the baseline measurement were included in the study. The county comprises 26 municipalities, from which 13 were included in the study and allocated into an intervention group of six municipalities and a comparison group of seven municipalities. 

Outlets were randomly selected by an external researcher from a register of outlets selling tobacco products, provided by the Stockholm County Administrative Board. Outlets were stratified by outlet type (i.e., grocery store, gas station, convenience store, and kiosk). Thus, the relative proportions of the outlet types in all municipalities were kept the same. The same outlets were visited at baseline and follow-up. After the baseline, municipal authorities were encouraged to perform compliance checks at outlets located in the intervention area, particularly the outlets included in the study. Municipal authorities in the comparison area were not informed about the study.

### 2.3. Procedures

Test purchases were conducted as previously described [41]. Adolescents were recruited in 2017 and 2019 through paper and digital advertisements at high schools in Stockholm County. Advertisements called 18-year-olds for an interview to work in a field study without stating the purpose of the study. In 2017, an expert panel consisting of persons who regularly meet adolescents through their profession or role (a teacher, nightclub doorman, social worker, and a child and adolescent psychiatrist) interviewed and rated the adolescents based on their perceived age and suitability for the study. The expert panel in 2019 consisted of a police officer, nurse from a youth substance use disorder clinic, and a youth football coach. Based on the scores, six females and three males were recruited in 2017, and three females and nine males in 2019. The adolescents had to sign a non-disclosure agreement to keep the performance of the study confidential. Additional research assistants were also recruited to drive the adolescents to the selected outlets. The adolescents and research assistants were instructed on how to conduct the test purchases before data collection. To avoid looking older, the adolescents were asked to wear neutral clothing (e.g., jeans and a t-shirt) and not use much make-up. These mystery shoppers entered the outlet in pairs consisting of one person attempting to purchase cigarettes and the other person discretely observing certain pre-defined characteristics, such as gender and age of the sales clerk and presence of age-limit signs regarding tobacco products. The role of shopper and observer was alternated for each attempt. The mystery shopper conducting the purchase attempt was instructed to ask for a pack of cigarettes of a certain brand and, if asked, state that they were 18-years old. If the clerk requested an ID, the mystery shopper was to search their pockets, then say that they had forgotten their ID and thereafter ask if they could purchase the cigarettes anyway. After the shopper and observer had left the outlet, a protocol was completed where the outcomes and characteristics were noted. 

### 2.4. Intervention

Civil servants from the authorities responsible for monitoring tobacco sales in the municipalities included in the intervention group were invited to a meeting in September 2018, to inform them about the study and encourage them to perform compliance checks in the outlets included at the baseline. At the meeting, the method of compliance checks—as described in the guide of the Public Health Agency of Sweden—was presented. The guide includes three steps to the method: planning, mystery shopping, and feedback. The content of the guide is based on the Alcohol Act (2010:1622), the Tobacco Act (1993:581), and the Public Health Agency of Sweden’s regulations and general advice on test purchases (FoHMS 2015:1). In brief, compliance checks are conducted in the following manner: A person who is aged 18-years old, but has a youthful appearance, is recruited as a mystery shopper, and a contract is established detailing the work and liability of this person and the authorities. The authorities inform the mystery shopper about the methodology and instruct them that they are to ask for a pack of cigarettes, but not show an ID. After each purchase attempt, the civil servant initiated a dialogue on the purchase outcome, legislation, and age-related routines, including ID checks, with the sales clerk and the outlet manager at the store and thereafter informs the store owner about the conducted test purchase and its outcome. The aim of the dialogue is to improve the outlet’s routines for age controls and compliance to legislations. 

After the follow-up, a process evaluation was conducted. All 13 municipalities were contacted and asked whether, when, and how compliance checks had been performed during the study period. Especially, municipalities were asked if they had conducted the compliance checks following the guidelines by the Public Health Agency of Sweden.

### 2.5. Outcomes

The primary outcome measure was refusal rates, defined as the proportion of attempts where a purchase was refused. The secondary outcome measure was the rate of ID requests, defined as the proportion of attempts where an ID was requested. These measures were assessed through covert test purchases at baseline in June 2017 and at follow-up in June 2019. 

### 2.6. Sample Size

**Figure 1:**
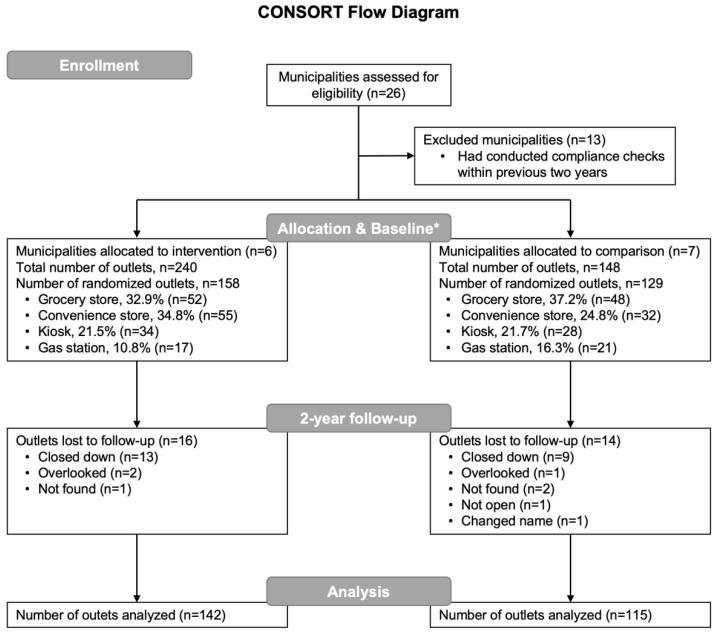
A flow diagram presenting the number of eligible and included municipalities as well as outlets at each assessment point. * A process evaluation conducted after the 2-year follow-up revealed that one municipality in the intervention group had not conducted the compliance checks intervention, while one municipality in the comparison group had conducted the intervention. These two municipalities hence changed group allocation. The final allocation of outlets is represented in the diagram.

An earlier Swedish study demonstrated a cigarette sale refusal rate of 52% [40] and we hypothesized that the compliance checks intervention would improve the refusal rate by 20%. Based on these assumptions, to detect a significant difference with at least 80% power (alpha = 5%, two-sided test), we needed at least 92 outlets in both groups (in total, 184 outlets). To allow for smaller improvements and dropout (e.g., closed outlets), we aimed to oversample and planned 150 purchase attempts in each group at baseline. In total, 300 outlets were planned to be visited at baseline and 20 outlets were selected as back-ups. Of these 320 outlets, purchase attempts could be conducted at 287 for reasons published previously [41]. At follow-up, the same 287 outlets were planned to be visited, resulting in test purchase attempts at 257 of these, (intervention group: *n* = 142, comparison group: *n* = 115), since the remaining outlets had closed down (*n* = 22), were overlooked (*n* = 3), not found (*n* = 3), not opened (*n* = 1), or had changed their name (*n* = 1) (Figure 1).

### 2.7. Data Analysis

Data were analyzed using the statistic software SPSS (IBM Corporation, Armonk, NY, USA, Version 25). For the outcome measures refusal rate (i.e., proportion of unsuccessful purchases) and the proportion of attempts when ID was requested, 95% confidence intervals according to the Jeffrey method [42] are reported. Descriptive statistics and Chi-square test were used to compare background characteristics between the intervention and comparison group at both time-points. The effect size (Cohen’s h) was calculated based on the proportions of the primary and secondary outcome measures, and interpreted as no (<0.20), small (0.20–0.49), medium (0.50–0.79), and big (≥0.80). For primary and secondary outcome measures, binary logistic regressions were used to test for significant effects of time, group, and time × group interaction, in order to investigate potential effects of the intervention and control for baseline factors. Bonferroni correction for multiple significance testing (0.05/3) was applied and *p* values < 0.017 were considered statistically significant. Evidence for non-significant findings were estimated using the Bayes factor and the following cut-offs were used: <1/3 evidence for null hypothesis, <1/3–1 equivocal evidence for null hypothesis, 1 no evidence, 1–3 anecdotal evidence for experimental hypothesis, >3 evidence for experimental hypothesis [43].

## 3. Results

### 3.1. Compliance Checks Conducted by Authorities

According to the original study design, six municipalities were allocated to the intervention and seven to the comparison group. Process evaluation revealed that out of the six intervention municipalities, five had performed compliance checks in accordance with the aforementioned guide. The remaining municipality, that had not conducted compliance checks was therefore moved to the comparison group. In four of the intervention municipalities, compliance checks were performed between spring and fall 2018, and in one municipality during spring 2019. In the comparison group, one municipality had performed compliance checks during spring 2019 in accordance with the guide from the Public Health Agency of Sweden, and was therefore moved to the intervention group for the analysis. Two other municipalities had conducted compliance checks but not in accordance with the guide. Furthermore, in one of these two municipalities, compliance checks were performed the month after the baseline measurement; that is, almost two years before the follow-up measurement. Therefore, these two municipalities were kept in the comparison group for the analysis. To clarify, since one municipality in the intervention group had not conducted compliance checks and one municipality in the comparison group had conducted compliance checks, these two municipalities changed group identity.

### 3.2. Characteristics of Comparison and Intervention Group

Based on the stratified selection, the most common outlet type was grocery stores, followed by convenience stores, kiosks, and gas stations, in both groups (Table 1). This distribution of outlet types did not differ between the comparison and intervention groups. There were no differences between groups at either baseline or follow-up regarding sex or age of the sales clerk or presence of 18-year age limit signs. At baseline, the proportion of female mystery shoppers was equal between both groups. At follow-up, no attempts were made by female purchasers in the comparison municipalities due to two reasons. First, few females were recruited and assessed to have a younger appearance at follow-up. Second, the original gender distribution between the control and intervention groups was changed after some municipalities had to change group identity, due to results of the process evaluation. While there was no difference between the groups in the proportion of attempts in which a colleague was near the sales clerk at baseline, this proportion was higher in the comparison group than in the intervention group at follow-up. 

### 3.3. Refusal Rates and ID Checks

Refusal rates of cigarette purchase attempts (primary outcome measure) and rates of ID checks (secondary outcome measure) did not significantly differ between the two study groups at baseline (Table 2). The refusal rates improved in both groups over time, albeit with no overall time effect (Table 3). There was evidence for an overall difference between the two groups as indicated by the Bayes factor of 6.6 (Table 3). A significant time × group interaction effect indicated that the rates in the two study groups differently changed over time. Although both groups improved, the change was greatest in the intervention group (Table 2). At follow-up, the two groups differed by 10.6 percentage units corresponding to a small effect size. ID checks followed a similar pattern to the refusal rates, but an improvement was only observed in the intervention group (Table 2). The difference between the two groups at the follow-up corresponded to a small effect size, and there was evidence for a time × group interaction effect (Table 3).

As shown in Table 2, the proportion of sales clerks in the intervention group who not only asked for ID during the follow-up, but also refused the youth purchase, was 95.8%. A closer analysis showed that of the 136 test purchases in which the mystery shopper was asked for an ID, purchase attempts were refused in 135 cases; that is, very good agreement (Cohen’s kappa = 0.83). 

## 4. Discussion

The present study aimed to investigate if compliance checks, consisting of information, mystery shopping, and feedback, are an effective method for decreasing the sales of cigarettes to underage adolescents. Results provide evidence that compliance checks performed by the municipality increased the refusal rates and rates of ID checks of pseudo-underaged mystery shoppers. Between baseline and follow-up, refusal rates of purchase attempts increased from 70.4% to 95.8% in the intervention group, but only from 80.0 to 85.2% in the comparison group. Furthermore, the rates of ID checks increased from 80.3% to 95.8% in the intervention group and did not change in the comparison group. For both outcomes, there was a significant group and time interaction effect, meaning that the refusal rates and rates of ID checks had increased at follow-up, and there was a difference in this regard between the two groups. Another interesting finding was that, in principle, all refusals had been preceded by the outlet clerks asking for ID. Thus, our results indicate that compliance checks effectively reduce cigarette sales rates to underage adolescents.

Previous studies have shown that active enforcement, involving sanctions such as fines, as well as multicomponent interventions involving information, education, dialogues with retailers, and media efforts, are more effective in reducing cigarette sales to adolescents than just giving information to retailers [9,12,13,14,20,21,22,23,24,25,26,27]. Nevertheless, in contrast to the present results, few studies achieved very high rates of ID checks [9,10,16,19,24]. An analysis of compliance checks conducted in the U.S. in 2017 and 2018 showed that although underaged adolescents were asked in 80% of the attempts to show an ID, tobacco products were sold in 9% of the attempts, and in 23% of these violations an ID had been asked for. Furthermore, violations were more likely if the underaged provided an ID [44]. This indicates that the true sales rates of tobacco products in Stockholm to underaged could be higher than the ones indicated by the present study. However, according to Swedish law, the municipality is not allowed to use underaged adolescents for compliance checks, and only the police are allowed to survey and sanctions such sales. In contrast, the Food and Drug Administration in the U.S. can use compliance checks to identify outlets that frequently sell to underaged individuals and issue no-tobacco-sale orders. A recent study showed that this sanction was underused among outlets selling repeatedly to underaged individuals and, in cases where it was used, could have been issued on average about a year earlier [45].

Compliance checks in accordance with the guide of the Public Health Agency of Sweden, which is based on the Tobacco Act, does not involve any sanctions. Instead, the method focuses on information and dialogue with the retailers. More specifically, outlets are first informed that compliance checks will be performed during a certain period. Mystery shopping is then conducted, followed by a dialogue with the sales clerk and the outlet manager, which is held by a civil servant working at the municipal authority responsible for controls on tobacco retailers. After the purchase attempts, the store owner is informed about the result of the purchase attempt, and information is given about the Tobacco Act and the importance of age controls. Previous studies have shown that feedback can effectively decrease cigarette sales to underage mystery shoppers. For example, a study conducted in 45 stores of a drug-store chain in three cities in the U.S., showed that monthly and bi-monthly compliance checks, including mystery shopping with feedback, successively increased ID checks from approximately 55–65% to approximately 85%. However, even in the comparison group, ID checks increased over time, potentially due to a spill-over effect [26]. Furthermore, compliance checks for alcohol, consisting of monthly mystery shopping, feedback to sales clerks or servers, and reports to managers increased rates of ID checks from 80% to 94–96% [46]. Similarly, an experimental study at 33 retail outlets using positive and negative feedback, as well as small rewards, also achieved very high compliance with ID checking upon alcohol purchases [47]. However, a recent small study with a quasi-experimental design conducted in Slovenia, revealed that the rather high sales rates of beer to underaged individuals and low frequency of ID checks improved to a small extent after the use of both undercover test purchases and compliance checks involving sanctions [48]. The relevance of alcohol compliance checks was recently demonstrated by a study in the U.S. showing that in times when many compliance checks were conducted, car crashes involving underaged drinking and driving were fewer [49]. Another study in Spain demonstrated that a multi-component intervention, including community mobilization and staff training, drastically increased the rate of ID checks when underaged adolescents attempted to buy alcohol [50]. In accordance with the first two alcohol studies described above, the present study demonstrates that compliance checks involving no sanctions can effectively induce very high compliance with ID checks and subsequent refusals upon tobacco purchase attempts. 

Refusal rates in most of the abovementioned studies [9,14,16,20,21,23,24,25,27] were lower at baseline (ranging from 11% to 60%) compared to our study. Since these studies were conducted many years ago, tobacco policies have since then become stricter worldwide, with increased age-limits for purchasing tobacco products in many countries, bans on smoking in bars and restaurants, and policies implemented to de-normalize smoking. Stricter policies and changing attitudes towards smoking could have contributed to the relatively higher baseline refusal rates in our study compared to earlier studies. The increase in refusal rates over time is also indicated by an earlier study conducted in three other regions in Sweden, where refusal rates were at an average 52% at the last measurement in 2005 [40]. On one hand, earlier studies on interventions had more room for improvement since refusal rates were lower than in the present study. On the other hand, de-normalization and changing attitudes could have facilitated a further improvement from an already high baseline regarding refusal rates through a greater willingness of sales clerks to comply with the legislation, once they had been reminded through compliance checks. Nevertheless, the finding that in about one in four attempts at baseline, adolescents were allowed to purchase cigarettes [42], can still be seen as a rather high availability and therefore, our results indicating that it was more difficult for adolescents to purchase cigarettes in the intervention municipalities have important implications. Future studies should investigate the effects of other methods that could help decrease sales rates of tobacco products to adolescents, such as automated ID requests and ID scanners, which have been recently shown to increase the compliance with ID checks [33,51]. Furthermore, a legal agreement between state and company, the so called “assurance of voluntary compliance”, used in various states and chains in the U.S. has also been shown to reduce retail violation rates. The precise form of the agreement varies, but can include training of employees, cash register programs to prompt ID checks for tobacco products, ID checks for shoppers appearing younger than 27, and mystery shopping programs [52]. Such a wide-reaching agreement could also be implemented in Sweden and their effects could be evaluated. 

### Strengths and Limitations

Strengths of the study are that outlets were randomly selected and the same outlets were visited at baseline and follow-up. Further strengths were the use of an expert panel to select adolescents who appeared underaged, as well as the use of a comparison area, where municipalities were not motivated to conduct compliance checks. 

A limitation of the study is that it did not use a randomized controlled trial design, since this is both difficult to conduct by civil servants in the municipalities, and spill-over effects between outlets are expected. Furthermore, right after the follow-up, a new Tobacco Act entered into force, which could have influenced the sales clerk behavior. Under this act, outlets must apply for permits from authorities responsible for controlling tobacco sales in the respective municipality. However, authorities cannot use compliance checks as a justification for injunctions, bans, revocation of licenses, or warnings. The act also banned smoking in certain outdoor locations, accessible to the public, such as entrances, train platforms, and outdoor cafés. Another limitation of the study is that no purchases were made by females in the comparison group at follow-up. In part, this was due to the switch of two municipalities between the intervention and the comparison groups, and also in part for not having recruited enough female mystery shoppers at the follow-up. However, since we have previously shown that females are refused purchase at a much lower rate [41], the difference between the groups might have been even larger if females had been employed in the comparison group. Another limitation is that no other tobacco products besides cigarettes were considered, as the use of other products (e.g., e-cigarettes) puts adolescents at greater risk of becoming daily cigarette smokers [53]. However, e-cigarettes are not as common as cigarettes among Swedish youth. Moreover, the study took place in 13 municipalities in the largest Swedish county (in terms of inhabitants), which poses a threat to generalizability. Finally, the fact that one municipality did not conduct compliance checks during the study period, despite encouragement to do so, further demonstrates that a general recommendation to conduct compliance checks might not be enough, as the municipalities would also need the means, resources, and motivation to prioritize such a measure. 

## 5. Conclusions

To prevent underaged adolescents from smoking and becoming adult smokers, the availability of cigarettes needs to be limited. Rather than implementing strong enforcement and sanctions, the methodology of compliance checks uses information about legislations and dialogue with outlet managers and sales clerks. Our results demonstrate that compliance checks, where sales clerks routinely ask young-looking customers for their ID, is effective in limiting availability by decreasing rates of cigarette sales to underaged adolescents. Since many Swedish municipalities already use compliance checks on a more or less regular basis, the method has the potential to play an important role in the tobacco prevention work in Sweden.

## Figures and Tables

**Table 1:** Characteristics of the comparison and intervention group at baseline and follow-up. At both baseline and follow-up, 257 test purchases were conducted at the same outlets. Of these purchase attempts, 142 were conducted in the intervention group (6 municipalities) and 115 in the comparison group (7 municipalities).

	Intervention % (*n*)	Comparison % (*n*)	Chi-Square (df), *p*-Value
Type of outlet			
Grocery store	34.5 (49)	40.9 (47)	4.58 (3), 0.205
Convenience store	33.8 (48)	24.3 (28)	
Kiosk	21.1 (30)	18.3 (21)	
Gas station	10.6 (15)	16.5 (19)	
Female shoppers			
Baseline	73.2 (104)	72.2 (83)	0.04 (1), 0.849
Follow-up	62.0 (88)	0	108.38 (1), <0.001
Female sales clerks			
Baseline ^1^	41.8 (59)	50.4 (58)	1.88 (1), 0.170
Follow-up	46.5 (66)	47.0 (54)	0.01 (1), 0.939
Sales clerks < 30 years			
Baseline	47.9 (68)	59.1 (68)	3.22 (1), 0.073
Follow-up	38.7 (55)	41.7 (48)	0.24 (1), 0.625
Colleague close by			
Baseline ^2^	44.7 (63)	41.7 (48)	0.22 (1), 0.637
Follow-up ^3^	37.9 53)	50.9 (58)	3.93 (1), 0.047
Age-limit sign			
Baseline	88.0 (125)	93.0 (107)	1.82 (1), 0.177
Follow-up	95.8 (136)	99.1 (114)	2.70 (1), 0.100

Data were missing for ^1^ *n* = 1; ^2^ *n* = 1; ^3^ *n* = 3.

**Table 2:** Results from test purchases in the intervention (*n* = 142) and comparison group (*n* = 115). The intervention group contains municipalities where compliance checks had been conducted according to the protocol of the Public Health Authority of Sweden during the study period (as revealed by the process evaluation).

	Intervention	Comparison	Difference between Proportions (95% CI)	Effect Size Cohen’s h
Refusal rate in % (95% CI ^1^), *n*				
Baseline	70.4 (62.6–77.5), 100	80.9 (73.0–87.2), 93	−10.5 (−1.5 to −22.5)	0.25
Follow-up	95.8 (91.5–98.2), 136	85.2 (77.9–90.8), 98	10.6 (2.8 to 18.4)	0.38
ID requests in % (95% CI), *n*				
Baseline	80.3 (73.2–86.2), 114	86.1 (78.9–91.5), 99	−5.8 (−4.2 to −15.8)	0.16
Follow-up	95.8 (91.5–98.2), 136	86.1 (78.9–91.5), 99	9.7 (2.1 to 17.3)	0.35

^1^ CI, confidence intervals.

**Table 3:** Binary logistic regression to adjust for baseline differences.

	Adjusted Odds Ratio (95% CI)	*p* Value ^1^	Bayes Factor ^2^
Refusal rate			
Time (follow-up vs. baseline)	1.36 (0.68–2.73)	0.381	1.5
Group (intervention vs. comparison)	0.56 (0.31–1.01)	0.056	6.6
Time × group interaction	6.98 (2.25–21.64)	<0.001	
ID requests			
Time (follow-up vs. baseline)	1.00 (0.47–2.11)	1.000	1.0
Group (intervention vs. comparison)	0.66 (0.34–1.29)	0.221	2.1
Time × group interaction	5.57 (1.71–18.16)	0.004	

^1^*p* values should be interpreted using the Bonferroni corrected values of <0.017. ^2^ Bayes factors were calculated for all non-significant independent variables.

## Data Availability

The data presented in this study are available on request from the corresponding author. The data are not publicly available due to ethical reasons.
